# Diatom-Derived Polyunsaturated Aldehydes Activate Cell Death in Human Cancer Cell Lines but Not Normal Cells

**DOI:** 10.1371/journal.pone.0101220

**Published:** 2014-07-03

**Authors:** Clementina Sansone, Alessandra Braca, Elena Ercolesi, Giovanna Romano, Anna Palumbo, Raffaella Casotti, Maria Francone, Adrianna Ianora

**Affiliations:** Stazione Zoologica Anton Dohrn, Naples, Italy; IISER-TVM, India

## Abstract

Diatoms are an important class of unicellular algae that produce bioactive polyunsaturated aldehydes (PUAs) that induce abortions or malformations in the offspring of invertebrates exposed to them during gestation. Here we compare the effects of the PUAs 2-*trans*,4-*trans*-decadienal (DD), 2-*trans*,4-*trans*-octadienal (OD) and 2-*trans*,4-*trans*-heptadienal (HD) on the adenocarcinoma cell lines lung A549 and colon COLO 205, and the normal lung/brunch epithelial BEAS-2B cell line. Using the viability MTT/Trypan blue assays, we show that PUAs have a toxic effect on both A549 and COLO 205 tumor cells but not BEAS-2B normal cells. DD was the strongest of the three PUAs tested, at all time-intervals considered, but HD was as strong as DD after 48 h. OD was the least active of the three PUAs. The effect of the three PUAs was somewhat stronger for A549 cells. We therefore studied the death signaling pathway activated in A549 showing that cells treated with DD activated Tumor Necrosis Factor Receptor 1 (TNFR1) and Fas Associated Death Domain (FADD) leading to necroptosis via caspase-3 without activating the survival pathway Receptor-Interacting Protein (RIP). The TNFR1/FADD/caspase pathway was also observed with OD, but only after 48 h. This was the only PUA that activated RIP, consistent with the finding that OD causes less damage to the cell compared to DD and HD. In contrast, cells treated with HD activated the Fas/FADD/caspase pathway. This is the first report that PUAs activate an extrinsic apoptotic machinery in contrast to other anticancer drugs that promote an intrinsic death pathway, without affecting the viability of normal cells from the same tissue type. These findings have interesting implications also from the ecological viewpoint considering that HD is one of the most common PUAs produced by diatoms.

## Introduction

Diatoms are microscopic, unicellular algae that represent dominant photosynthetic organisms in the world's oceans. Their beneficial role as food for grazers was challenged over a decade ago after the discovery that some diatom species produce teratogenic compounds such as polyunsaturated aldehydes (PUAs) that induce abortions, birth defects, poor development and high offspring mortality in predatory planktonic and benthic invertebrates [1]–[3]. PUAs are the end-products of a lipoxygenase/hydroperoxide lyase metabolic pathway [4] initiated by damage to algal cells, as occurs through grazing by predators. Cell damage activates lipase enzymes which liberate polyunsaturated fatty acids (PUFAs) from cell membranes that are immediately oxidized and cleaved within seconds to form PUAs and a plethora of other compounds collectively termed oxylipins [4], [5]. Numerous functions have been proposed for PUAs such as: grazer defense [6], [7]; allelopathy [8], [9], cell to cell signaling [10], antibacterial activity [11], [12], and bloom termination [13], [14]. Thus, the same secondary metabolites have multiple functions from grazing defense to signal molecules mediating several plankton interactions.

First described in marine diatoms by Miralto et al. [15] who showed that the PUAs 2-*trans,*4-*cis*,7-*cis*-decatrienal, 2-*trans*,4-*trans*,7-*cis*-decatrienal and 2-*trans*,4,*trans*-decadienal (DD) arrested embryonic development in copepods and sea urchins, and had antiproliferative and apoptotic effects on the human adenocarcinoma CaCo2 cell line. Successively, both laboratory and field studies have demonstrated a detrimental impact of diatom diets on copepod grazer reproductive success [6], [16]–[19]. PUAs may be sequestered during oocyte development and be passed maternally to the embryo, or may act directly on embryos. By whichever route, the timing of reproduction in relation to toxic diatom abundance will have important consequences for invertebrate recruitment [6].

Similar compounds are produced by higher flowering plants that are believed to play a pivotal role in plant defense because they act as chemical attractors (e.g. pheromones, pollinator attraction) or alarm signals against herbivore attack (e.g. in tritrophic interactions) and protective compounds (antibacterial, wound healing) [20]. Diatom oxylipins also show a high similarity to volatile organic compounds released from brown algae which are suggested to be involved in chemical signaling and pheromone attraction between gametes of different sex [20] and seem to be intermediates for innate immunity [21].

The PUA DD is the best-studied metabolite of this group and has thus become a model aldehyde for experimental studies on the effects of oxylipins on marine organisms (reviewed in [1], [3]). However, several marine diatoms have been shown to produce PUAs other than DD, including *Skeletonema marinoi*, a cosmopolitan bloom forming diatom that produces 2-*trans*,4-*trans* -heptadienal (HD), 2-*trans*,4-*trans* -octadienal (OD) and 2-*trans*,4-*trans*,7-octatrienal (octatrienal) [22], [23]. Of these, the most common is HD [13], [24]. Ceballos and Ianora [25] conducted experiments testing the effects of DD, OD and HD on copepod egg hatching success showing that the longer the chain length of the PUAs, the stronger the biological activity of these molecules, as also confirmed by [26] on bacteria, algae, fungi, echinoderms, molluscs and crustaceans, and [27] using sea urchin eggs. Ribalet et al. [9] also found a concentration-dependent reduction in the growth rate of marine phytoplankton, belonging to different taxonomic groups, exposed to PUAs with longer-chained aldehydes having stronger effects on growth than shorter-chained aldehydes.

Here we compare the effects of different PUAs, including DD, OD, and HD on the lung adenocarcinoma cell line A549, colon adenocarcinoma derived from metastatic ascites COLO 205 cell line, and the lung/brunch normal epithelial cell line BEAS-2B. PUAs are known to cause apoptosis [15], [28]-[29] but in a previous study [6] they were shown to exert dramatic effects only on proliferating, undifferentiated cells. We therefore used two highly aggressive tumor cell lines to better understand the pathways involved in cell death and a normal one to assess a hypothetical specific action against proliferating cell types. Another major objective was to compare the effects of DD, a model PUA in toxicological experiments but not the most common PUA present in marine phytoplankton (in a survey of 51 species of marine diatoms, DD was the least detected PUA, [24]) with the more prevalent diatom PUAs HD and OD. These are more likely to cause insidious (sensu [15]) antiproliferative effects on planktonic and benthic grazers *in situ* during natural diatom blooms such as those reported by [3] and references therein.

## Material and Methods

### Cell cultures and treatment

The A549 (ATCC CCL185) human lung adenocarcinoma and COLO 205 (ATCC CCL-222) colon adenocarcinoma metastatic ascites-deriving cell lines were maintained in DMEM (Dulbecco's modified Eagle's medium) supplemented with 10% fetal bovine serum (FBS), 100 units ml^−1^ penicillin and 100 µg ml^−1^ streptomycin. Cells were incubated in a 5% CO_2_ humidified chamber at 37°C for growth. A549 and COLO 205 cells (2×10^4^ cells well^−1^) were seeded in a 24-well plate and kept overnight for attachment. The next day the medium was replaced with fresh medium with three concentrations (2, 5 and 10 µM) for each of three PUAs (DD, OD, and HD, Sigma-Aldrich Inc., Milano, Italy) tested; cells were allowed to grow for 24, 48 and 72 h. After incubation, the supernatant was removed and adherent cells were examined for viability. A549 cells used for protein/RNA extraction and cell cycle analysis 2×10^6^ were seeded in Petri dishes (90 mm diameter) and treated as reported above.

In an independent experiment, A549 cells (2×10^3^ cells well-1) were seeded in a 96-well plate and kept overnight for attachment. The next day the medium was replaced with fresh medium with three concentrations (2, 5 and 10 µM) for each of three PUAs (DD, OD, and HD, Sigma-Aldrich Inc., Milano, Italy) tested and with caspase-3 Inhibitor (C_30_H_41_FN_4_O_12_, sc-3075, Santa Cruz) at 9.7 µM; cells were allowed to grow for 24, 48 and 72 h. After aldehyde treatment, viable cells were evaluated as described below. The BEAS-2B (ATCC CRL-9609) lung/brunch normal epithelial cell line was maintained in DMEM (Dulbecco's modified Eagle's medium) supplemented with 50% fetal bovine serum (FBS), 100 units ml^−1^ penicillin and 100 µg ml^−1^ streptomycin. Cells were incubated in a 5% CO_2_ humidified chamber at 37°C for growth. BEAS-2B (2×10^3^ cells well^−1^) was seeded in a 96-well plate and kept overnight for attachment. The next day the medium was replaced with fresh medium with three concentrations (2, 5 and 10 µM) for each of three PUAs (DD, OD, and HD, Sigma-Aldrich Inc., Milano, Italy) tested; cells were allowed to grow for 24, 48 and 72 h. After incubation, the supernatant was removed and adherent cells were examined for viability.

### Viability assays

We performed two types of viability assays: MTT and Trypan blue assay. We here choose to represent the most significant data obtained with one or the other type of test depending on the characteristics of the treated cells. In particular normal cells (BEAS-2B) that were not affected by PUAs treatment (and hence there were no dead cells) were examined with the MTT colorimetric assay whereas A549 and COLO 205 cells were colored with trypan blue which stains only dead cells. Furthermore, A549 cells treated with PUAs in the presence of caspase-3 inhibitor were also examined with the MTT assay to assess inhibition of toxicity.

For Trypan blue, A549 and COLO 205 cells (2×10^4^/well) were seeded in each well of a 24-well plate and kept overnight for attachment in the presence of Dulbecco's medium. The next day, the medium was replaced with fresh medium containing 0, 2, 5 or 10 µM of DD, OD or HD. Treated cells were incubated for 24, 48 and 72 h. Following incubation, the supernatant was collected and discarded, while adherent cells were treated with a 0.4% trypan blue solution (Hyclone, Lot no: JRH27098) according to the Trypan Blue Dye Exclusion assay [30]. After coloring, cells were detached with trypsin, centrifuged, and the pellet washed with Phosphate buffer saline (PBS); 10 µl of this solution was placed in a Burker counting chamber. Blue cells (indicating dead cells) were counted in each area and compared to controls to calculate % cell viability.

For MTT, A549 and BEAS2B cells were seeded in 96-well plate (2×10^3^ cells/well), after treatment times, and were incubated with 10 µl (10 mg/ml) of MTT (3-[4,5-methylthiazol-2yl]-2,5-diphenyl-tetrazoliumbromide, Applichem A2231). The number of viable cells after aldehyde (DD, OD, HD) treatment was evaluated by spectrophotometric MTT assay according to the manufacturer's protocol and calculated as the ratio between mean absorbance (λ = 570 nm) of sample and mean absorbance of control and expressed as percentage viability.

### Acridine orange/ethidium bromide double staining test for morphological analysis

Control and treated adherent A549 cells were trypsinized and collected by centrifugation at 500 g for 5 min. Cells were washed 3× with PBS and changes in cell morphology were detected with the acridine orange/ethidium bromide staining test. Cells were re-suspended in 25 µl of dye (100 µg ml^−1^ of acridine orange and 100 µg ml^−1^ of ethidium bromide prepared in PBS and gently mixed). 10 µl of dyed cells were placed on a microscope slide, covered with a coverslip and examined under a confocal microscope (Zeiss LSM510, laser 488 with LP505 filter for green fluorescence; laser 543 with LP 560 filter for red fluorescence) with 25× objective. Acridine orange penetrates both living and dead cells emitting green fluorescence when intercalated into normal double-stranded nucleic acids (DNA) and red fluorescence when bound with damaged single-stranded nucleic acids. Ethidium bromide penetrates only dead cells with damaged membranes emitting red fluorescence. Four cell types were identified according to [31] on the basis of fluorescence emission and morphological aspect of chromatin condensation in stained nuclei: (1) viable cells with uniformly bright green nuclei and an organized structure (control); (2) early apoptotic cells with irregular green nuclei with condensed chromatin and with apoptotic bodies stained in red, (3) late apoptotic cells with orange to red nuclei with highly fragmented chromatin; (4) uniformly orange to red nuclei with an organized structure ascribable to necrotic cells.

### RNA isolation and RT^2^ Profiler PCR Arrays

After treatment, A549 cells were washed in tissue culture dish by adding cold PBS and rocking gently. Cells were washed directly in a culture dish by adding 1 ml of Trizol Reagent (Life technologies, cat. 10296–010) per 10 cm diameter dish and scraping with cell scraper. RNA was isolated according to the manufacturer's protocol. RNA concentration and purity was assessed using the nanophotomer NanodroP (Euroclone). RNA (400 ng) was subjected to reverse transcription reaction using the RT^2^ first strand kit (Qiagen, cat.330401) according to the manufacturer's protocol.

To assess the expression of apoptotic genes, real-time quantitative reverse transcription PCR (qRT-PCR) was performed using the RT^2^ Profiler PCR Arrays kit (Qiagen, cat.330231). Experiments were performed in triplicates for A549 cell line treated with PUAs at 5 µM concentration for 2 hours exposure time.

Plates were run on a ViiA 7 (Applied Biosystems 384 well blocks), Standard Fast PCR Cycling protocol with 10 µl reaction volumes. Cycling conditions used were – 1 cycle initiation at 95.0°C for 10 min and followed by amplification for 40 cycles at 95.0°C for 15 s and 60.0°C for 1 min. Amplification data were collected via ViiA 7 RUO Software (Applied Biosystems). The Ct-values were analyzed with PCR array data analysis online software (http://pcrdataanalysis.sabiosciences.com/pcr/arrayanalysis.php, Qiagen).

### Protein extraction

A549 cell lysate, after treatment, was prepared by scraping the cells of each Petri dish into 1 ml of RIPA lysis buffer (150 mM NaCl, 50 mM Tris-HCl pH 7.6, 5 mM EDTA, 0.5% NP-40, 0.5% sodium deoxycholate, 0.1% SDS) supplemented with protease inhibitors (1 µM PMSF and Complete Protease Inhibitor Cocktail Tablets, Roche, Monza, Italy) and phosphatase inhibitors (PhosSTOP Cocktail Tables, Roche). The lysate was incubated on ice for 15 min and then clarified by centrifugation at 14,000 g for 20 min. Total protein concentration was determined using a Bio-Rad Protein Assay Reagent (Bio-Rad, Milan, Italy) with bovine serum albumin (BSA) as a standard. The protein extract was stored at – 80°C until use.

### Electrophoresis

Before electrophoresis, protein samples were incubated at 100°C for 5 min. Following 10% SDS-PAGE, gels were stained with Coomassie or blotted onto nitrocellulose (Hybond, GE Healthcare) membrane. Membranes were blocked for 1 h in 1X Tris Buffered Saline (TBS), with 0.1% Tween-20 with 5% w/v nonfat dry milk, and incubated overnight at 4°C with the primary antibodies diluted in 1X TBS, 0.1% Tween-20 with 5% BSA. Primary antibodies were from the Death Receptor Antibody Sampler Kit (Cell Signaling Technology, 8356S) including anti-TNFR1 (1∶1000), anti-TNFR2 (1∶1000), anti-FADD (1∶1000), anti-RIP (1∶1000), anti-Fas/FasL (1∶1000). Anti-active Cas-3 (1∶1000) purchased from BioVision. Positive control was obtained by using anti-actin antibody (1∶500) (Sigma). Actin was used as a control for each protein analysed. Here we show only a representative gel for actin. After incubation, membranes were washed three times for 5 min each with 15 ml of TBS/Tween and then incubated with HRP-conjugated secondary antibody anti-rabbit (1∶2000, Cell Signaling Technology) with gentle agitation for 1 hour at room temperature. After incubation, membranes were washed three times for 5 min each with 15 ml of TBS/Tween. Blotted membranes were immunodetected using ECL Prime western blotting detection reagent (GE Healthcare). Proteins were visualized with Amersham hyperfilm (GE Healthcare). Densitometric analysis of immunopositive bands was performed using Image J software.

### Cell cycle analysis

A549 cells were collected from plates using 1 mL of Trypsin-EDTA (Lonza, Italy), fixed in 70% ethanol and stored at −20°C. Cells were then washed twice with PBS, resuspended in PBS containing 1 mg ml^−1^ RNase A (Qiagen, Cat.19101), incubated at 37°C for 45 min and then stained with propidium iodide (PI, 10 µg ml^−1^) for 15 min. The DNA distribution inside cells was then estimated using a FACScalibur flow cytometer (BD Biosciences Immunocytometry Systems, San Jose, CA, USA). The percentage of cells in the different phases of the cell cycle was calculated using ModFit LT (Verity Software House, Topsham, ME, USA).

### Statistical analysis

Statistical differences between treated and control cells for cell viability counts were determined by One-way ANOVA and Dunn's Multiple Comparison Test with significant p values ≤0.05 using GraphPad Prism (GraphPad Software, San Diego California USA).

Gene expression data were analyzed by PCR array data analysis online software (http://pcrdataanalysis.sabiosciences.com/pcr/arrayanalysis.php, Qiagen). The histograms show relative expression ratios of the analyzed genes with respect to controls without PUAs. Only expression values greater than a 1.55-fold difference with respect to the controls were considered significant.

Immunoblotting protein expression was calculated as the percentage of integral area of every single gel band with respect to total gel lane area, represented as pixels. Statistical differences between treated and controls were determined by T-student analysis with significant p values ≤0.05. Data significantly different from controls, with p values <0.001 are marked with 2 asterisks in the figures.

## Results

### Viability of A549 and COLO 205 cancer cell lines, and normal BEAS-2B cell line after treatment with the PUA decadienal (DD)

**Figure 1:**
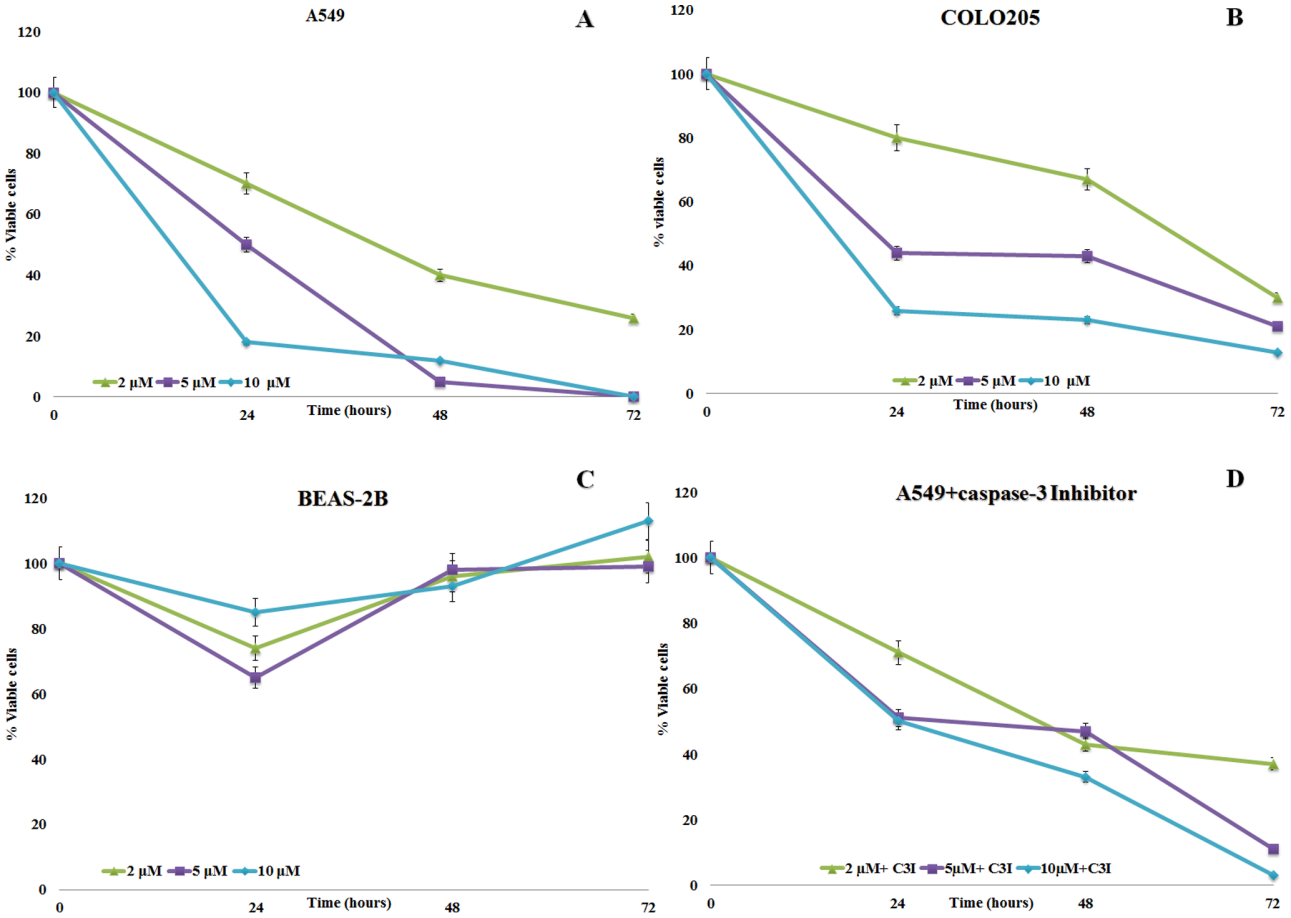
(A, B, C) Effect of the diatom PUA 2-*trans*,4-*trans*-decadienal (DD) on the human lung adenocarcinoma cell lines A549 and COLO 205, and the lung/brunch normal epithelial BEAS-2B cell line. (D) Effect of DD on A549 cell line in the presence of caspase-3 inhibitor (9.7 µM). Percentage of viable cells for A549 and COLO 205 calculated with the Trypan blue viability assay and for BEAS-2B with the MTT viability assay. Values are reported as mean ±S.D compared to controls (100% viability); ▴ 2 µM; ▪ 5 µM, ♦ 10 µM.

As shown in Fig. 1A–1B, treatment of A549 and COLO 205 cells with 2, 5 or 10 µM of DD resulted in a significant dose- and time-dependent reduction in cell viability compared to controls (p<0.05). After 24 h, a decrease in the percentage of viable A549 cells was observed with all doses tested (70%, 50% and 18% viable cells at 2, 5 and 10 µM DD concentrations, respectively). Similar results were obtained with COLO 205 cells (80%, 44% and 26% viable cells at 2, 5 and 10 µM DD concentrations, respectively). After 48 h of treatment, a further reduction was observed, especially at higher DD concentrations of 5 and 10 µM for A549 cells; COLO 205 viability diminished more slowly (67%, 43% and 23% viable cells at 2, 5 and 10 µM DD concentrations, respectively). At 10 µM A549 cells showed evident morphological alterations, such as the loss of contact adherence with other cells as well as with the plate substrate. After 72 h, A549 cell viability decreased to 0% with 5 and 10 µM DD and to 26% with 2 µM DD; COLO-205 cell viability was 30%, 21% and 13% with 2, 5 and 10 µM DD, respectively.

Treatment of BEAS-2B cells with 2, 5 or 10 µM of DD did not significantly reduce cell viability compared to controls (Fig. 1C). After 24 h DD treatment BEAS-2B cell viability decreased slightly (74%, 65% and 85% viable cells at 2, 5 and 10 µM DD concentrations, respectively), but after 48 and 72 h cell viability recovered and was comparable to controls, indicating mild toxic effects after 24 h only. Finally, to assess the specific cytotoxic effect recorded for the A549 cell line, the MTT assay experiment was performed in the presence of a caspase-3 inhibitor for all aldehyde treatments. Figure 1D shows that the percentage of cell viability is comparable to cells treated with PUAs without inhibitor at 2 µM; at 5 and 10 µM there is a slower decrease in cell viability compared with treatment without caspase-3 inhibitor after 48 h (Fig.1A), but after 72 h the same concentrations induced an increase in cell death.

### Viability of A549 and COLO 205 cancer cell lines and normal BEAS-2B cell line after treatment with the PUA octadienal (OD)

**Figure 2:**
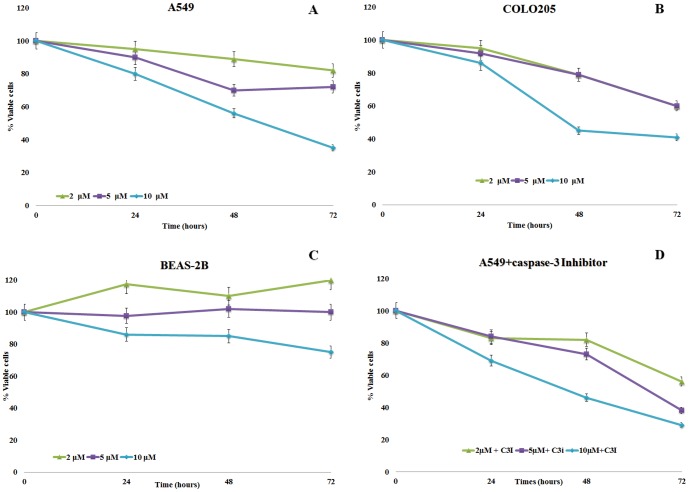
(A, B, C) Effect of the diatom PUA 2-*trans*,4-*trans*-octadienal (OD) on the human lung adenocarcinoma cell lines A549 and COLO 205, and the lung/brunch normal epithelial BEAS-2B cell line. (D) Effect of OD on A549 cell line in the presence of caspase-3 inhibitor (9.7 µM). Percentage of viable cells for A549 and COLO 205 calculated with the Trypan blue viability assay and for BEAS-2B with the MTT viability assay. Values are reported as mean ±S.D compared to controls (100% viability); ▴ 2 µM; ▪ 5 µM, ♦ 10 µM.

Treatment of A549 cells with 2, 5 or 10 µM of OD inhibited cell proliferation with time, especially at higher concentrations (10 µM) when cell viability decreased to 35% after 72 h (Fig. 2A). At 2 µM concentrations, the effect on cell viability after 24 h was not significantly different compared to controls, but became so after 72 h (p<0.05). Whereas COLO 205 cell viability after 24 h did not decrease significantly (Fig. 2B), cytotoxic effects became stronger after 72 h for all three OD concentrations (60%, 60% and 41% viable cells at 2, 5 and 10 µM OD concentrations, respectively, p<0.05).

Treatment of BEAS-2B cells with 2 and 5 µM of OD did not significantly reduce cell viability compared to controls after 24, 48 and 72 h (Fig. 2C), whereas at 10 µM OD, BEAS-2B cell viability decreased slightly after 72 h (75% viable cells). Treatment of A549 cells with OD in the presence of caspase-3 inhibitor did not result in significant differences in percentage cell viability (Fig. 2D).

### Viability of A549 and COLO 205 cancer cell lines and normal BEAS-2B cell line after treatment with the PUA heptadienal (HD)

**Figure 3:**
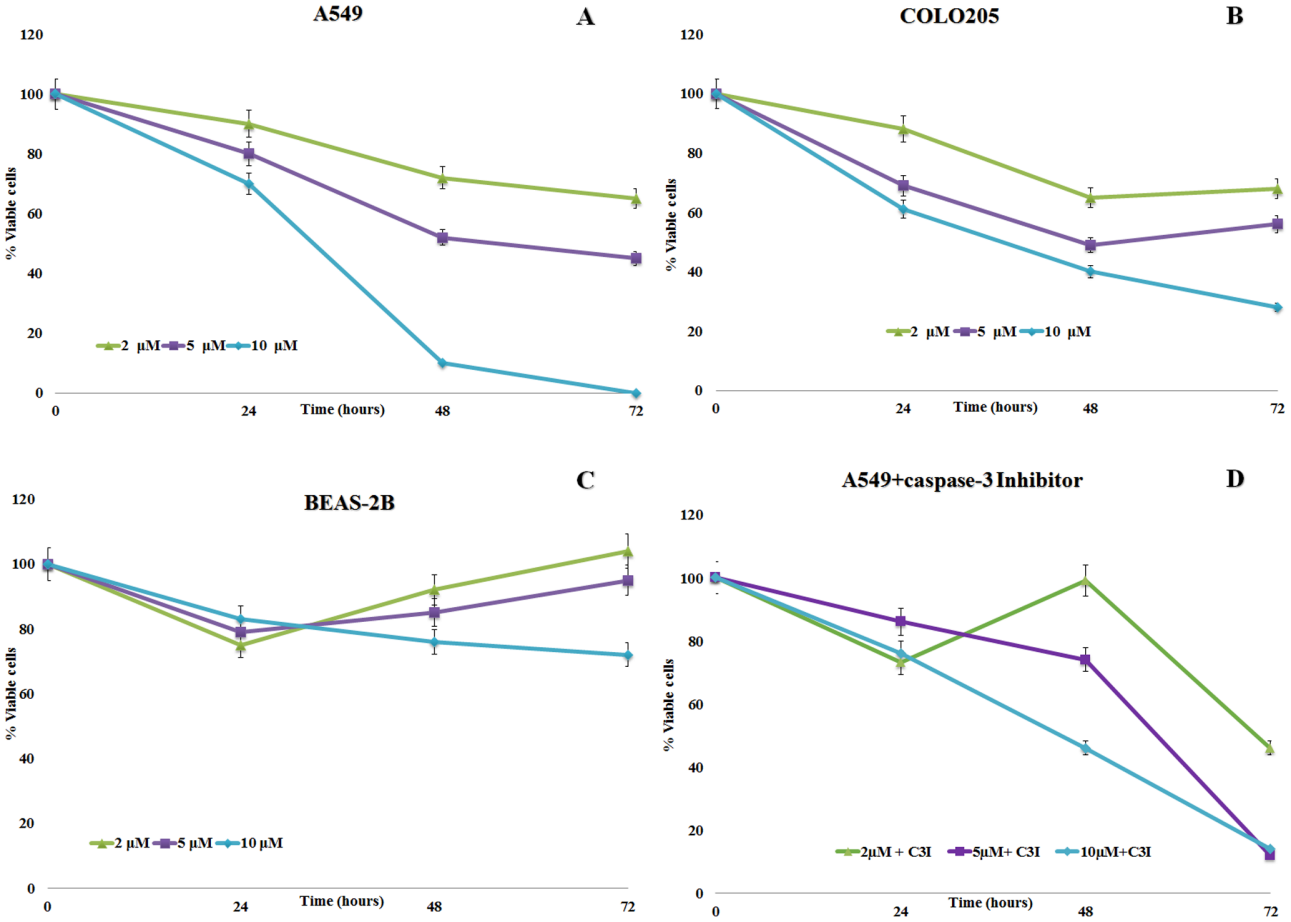
(A, B, C) Effect of the diatom PUA 2-*trans*,4-*trans*-heptadienal (HD) on the human lung adenocarcinoma cell lines A549 and COLO 205, and the lung/brunch normal epithelial BEAS-2B cell line. (D) Effect of HD on A549 cell line in the presence of caspase-3 inhibitor (9.7 µM). Percentage of viable cells for A549 and COLO 205 calculated with the Trypan blue viability assay and for BEAS-2B with the MTT viability assay. Values are reported as mean ±S.D compared to controls (100% viability); ▴ 2 µM; ▪ 5 µM, ♦ 10 µM.

As shown in Fig. 3A, treatment with 2, 5 and 10 µM of HD on A549 cells slightly inhibited cell proliferation after 24 h but the effect was still significantly different with respect to controls (p<0.05). The effect was more evident with time. In particular, at 10 µM only 10% of cells were viable after 48 h and 0% after 72 h. HD treatment on COLO 205 cells also caused a reduction in cell viability, but the effect was not as strong as with A549 cells. After 48 and 72 h (Fig. 3B), a significantly toxic effect was observed at high HD concentrations (40% and 28% cell viability at 10 µM HD).

As in the case of DD and OD, treatment of BEAS-2B cells with HD did not induce toxicity (Fig. 3C). Treatment of A549 cells with HD in the presence of caspase-3 inhibitor did not result in differences in percentage of viable cells at higher HD concentrations (5 and 10 µM) (Fig. 3D). At 2 µM, there was a significant increase in cell mortality in the presence of caspase-3 Inhibitor after 72 h (Fig.3D).

### Morphological effects of the diatom PUAs DD, OD and HD on A549 cell line

**Figure 4:**
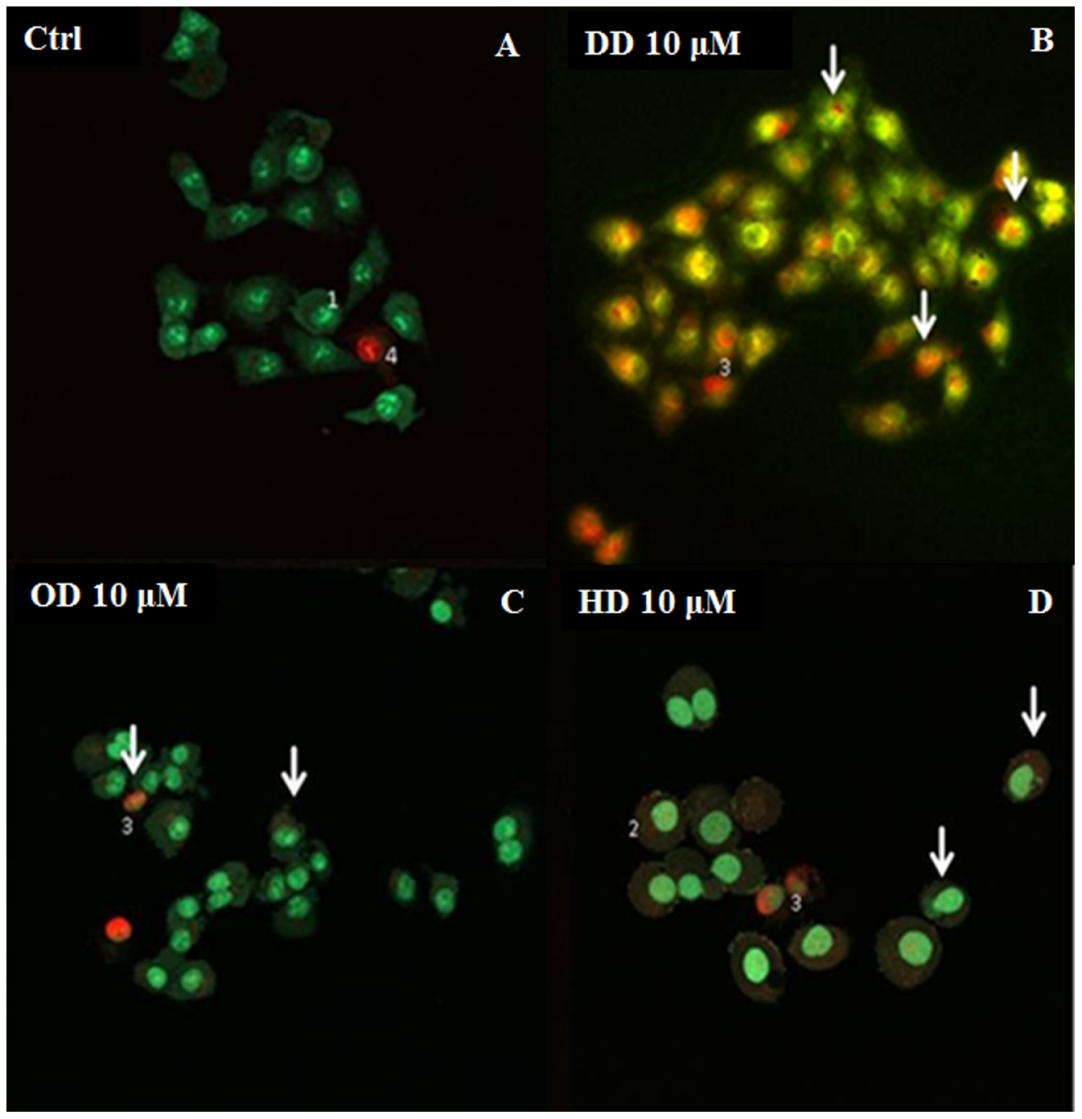
Effect of the diatom PUAs 2-*trans*,4-*trans*-decadienal (DD), 2-*trans*,4-*trans*-octadienal (OD) and 2-*trans*,4-*trans*-heptadienal (HD) on the human lung adenocarcinoma cell line A549. Control and treated cells double stained with acridine orange and ethidium bromide after 48 µM DD, OD and HD observed at the confocal microscope. Numbers indicate (1) normal cells; (2) early apoptotic cells; (3) late apoptotic cells; (4) necrotic cells (see material and methods for details). Arrows indicate cells with fragmented nuclei.

To observe morphological changes in cells treated with DD, OD and HD and to verify if decreased viability after treatment was correlated with apoptosis induction, we double stained cells with the fluorescent dyes for nucleic acids, acridine orange (AO) and ethidium bromide (EO) after 48 h of treatment. Viable cells were identified by bright green nuclei in intact cells (AO). Early apoptotic cells were identified by irregularly structured green nuclei with condensed chromatin and orange or light red patches. Late apoptotic cells contained positively stained nuclei with both dyes (EB and AO), appearing orange or light red together with apoptotic bodies. Nuclei of necrotic cells had intact chromatin and appeared red. All control nuclei appeared green with a regular spherical structure and chromatin organization except for a few senescent cells in a necrotic stage that showed red fluorescent nuclei (Fig. 4A). At 10 µM DD, all cells were in the late apoptosis stage and were characterized by yellow-orange double staining (AO/EB) due to chromatin condensation and loss of membrane integrity (Fig. 4B). At 10 µM OD, cell damage was less evident with distinct changes in morphology and the presence of red staining in some cells, indicating progression in cell apoptosis (arrows in Fig. 4C). At 10 µM HD, cell morphology was substantially altered with dispersed chromatin in the nuclei which appeared enlarged. Hallmarks of late apoptosis such as nuclear fragmentation were also visible (arrows in Fig. 4D).

### Immunoblot analyses of death receptors (TNFR1, TNFR2, RIP, FADD) and effectors (caspase-3) on A549 cell line treated with PUAs (DD, OD and HD)

**Figure 5:**
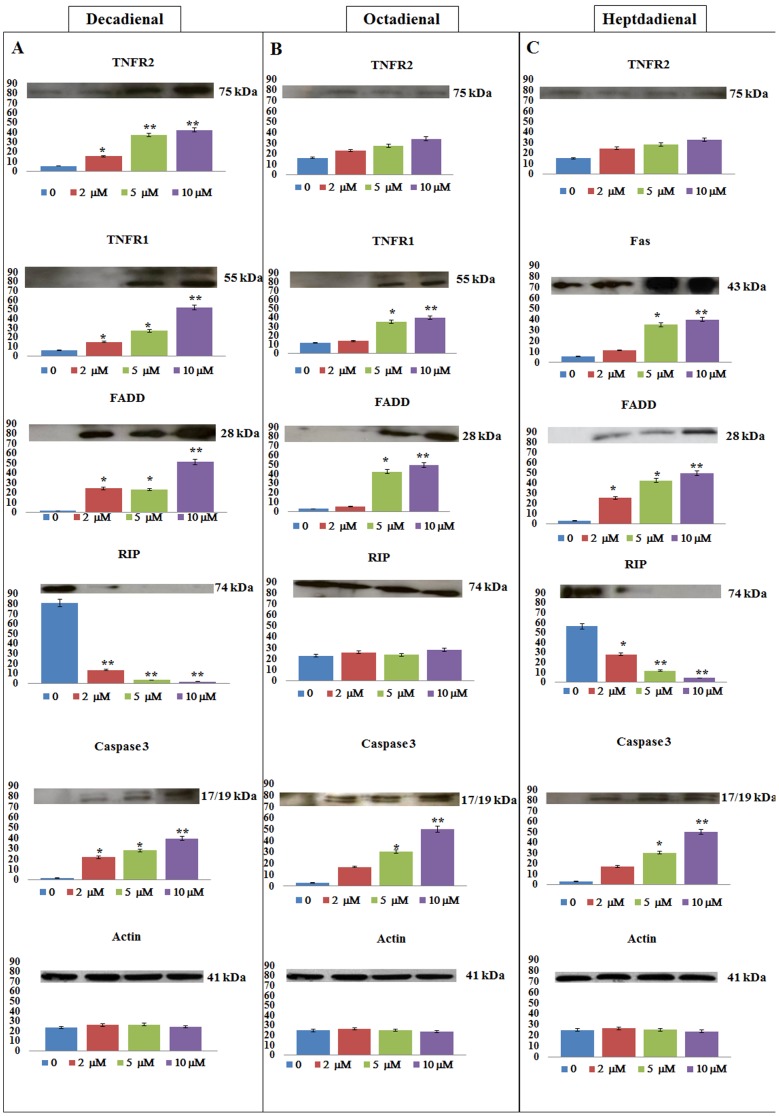
The histograms show the effects of 2-*trans*,4-*trans*-decadienal (DD), 2-*trans*,4-*trans*-octadienal (OD) and 2-*trans*,4-*trans*-heptadienal (HD) on the expression levels of target proteins (TNFR2, TNFR1, Fas, FADD, RIP, caspase-3) and control (actin) in lung adenocarcinoma A549 cells. Immunoblot analysis shows that PUAs induce TNF signaling after 24(DD and HD) and 48 h (OD) of treatment with actin. Asterisk denotes significant increase in protein levels measured. **p≤0.05 versus control; error bars represent ±SD.

After 24 h of treatment, DD induced a dose-dependent increase in the expression of Tumor Necrosis Factor Receptor 2 (TNFR2) especially at 5 and 10 µM, compared to controls, as revealed by densitometric analysis of the photographic sheet (Fig. 5A) of the immunoblotting membrane. On the contrary, TNF receptor associated factors (TRAF1 and TRAF2) were not activated indicating the absence of a survival pathway. Moreover, DD induced an activation of TNFR1 in cells treated with 5 and 10 µM concentrations (Fig. 5A). The downstream Fas-Associated protein with Death Domain (FADD) was also strongly activated at 10 µM concentrations, while levels of the Receptor-interacting Protein (RIP) decreased dramatically at all three DD concentrations (Fig. 5A). Reduction in the expression of RIP represents an early death signaling pathway as indicated by the absence of adaptor proteins such as Tumor necrosis factor Receptor type 1-Associated Death Domain protein (TRADD) or TNF Receptor Associated Factors TRAF1 and TRAF2 (data not shown). DD also activated caspase-3 confirming cell death via apoptosis after 24 h (Fig. 5A). Other death receptors implicated in the extrinsic apoptotic pathway (Fas, DR3, DR4, DR5) as well as some factors activated in intrinsic apoptotic pathways (Akt1, Akt2, PARP, Apaf1) were not involved in the cell response to DD (data not shown).

The same pathway was observed after OD treatment but only after 48 h. TNFR2 expression slightly increased compared to controls but the difference was not significant. TNFR1 and FADD showed a significant response at the highest concentrations (5 and 10 µM) (Fig. 5B). In contrast with DD, OD-treated cells showed a strong and persistent expression of RIP, indicating the coexistence of a survival and death signaling response (Fig. 5B). Caspase-3 was also activated as with DD (Fig. 5B).

HD did not significantly increase the expression of TNFR2 (Fig. 5C) and did not trigger any response in TNFR1 after 24 h and 48 h. After 24 h HD strongly increased the expression of Fas receptor (Fas/FasL-Ligand System) in a dose-dependent manner. Maximum effect of HD on Fas was observed at 5 and 10 µM concentrations (Fig. 5C). HD also increased FADD expression and activated caspase-3 (Fig. 5C). By contrast, levels of RIP decreased dramatically after 24 h (Fig. 5C). Moreover, Apaf1 was not revealed after HD treatment at 24 and 48 h (Data not shown).

### Expression analysis of genes encoding for death receptors (TNFR1, TNFR2, RIP, FADD) and effectors (Caspase-3, AIFM1) on A549 cell line treated with PUAs (DD, OD and HD)

To better understand the toxic effects at the molecular level, A549 cells were analyzed after 2 h of PUAs treatment because all proteins for DD and HD were already expressed and activated after 24 h. We chose to use 5 µM concentration since at higher concentrations effects were too severe.

**Figure 6:**
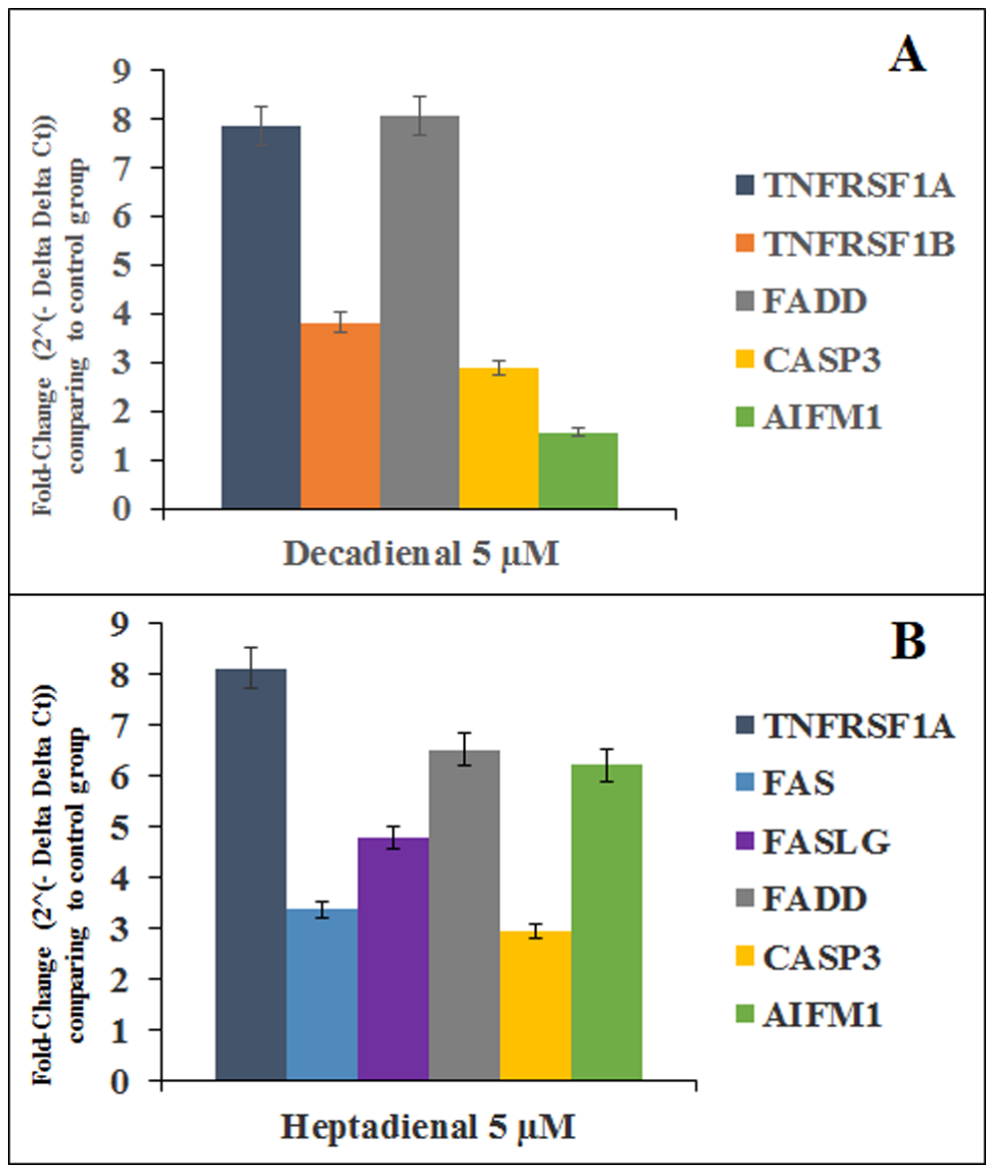
Histograms show the effects of 2-*trans*,4-*trans*-decadienal (DD) and 2-*trans*,4-*trans*-heptadienal (HD) on the expression levels of target genes in lung adenocarcinoma A549 cells. Gene expression analysis was conducted after 2 µM DD and HD; error bars represent ±SD.

Control genes for real-time qPCR were Actin-beta (ACTB), Beta-2-microglobulin (B2M), Hypoxanthine phosphoribosyltransferase (HPRT1) and Ribosomal protein large (RPLP0), the expression of which remained constant in A549 cells. The histograms reported in Figure 6 show the relative expression ratios of the analyzed genes with respect to controls without PUAs. Only expression values greater than a two-fold difference with respect to the controls were considered significant. Figure 6 shows expression levels of genes that were directly correlated with activation of corresponding proteins analyzed by western blotting.

After 2 h of treatment of DD at 5 µM, there was an up-regulation of the *tumor necrosis factor receptor superfamily TNFRSF1A* (7.86- fold change) and *TNFRSF1B* (3.82-fold change) that code for the two receptors TNFR1 and TNFR2 (Fig. 6A) as revealed by protein analysis (Fig 5A). *FADD* was also strongly up-regulated (8.06-fold change) (Fig. 6A). C*aspase-3* and *AIFM1* (*Apoptosis-Inducing Factor 1)* were up-regulated after 2 h treatment with 5 µM DD (1,8741 and 1.65-fold change) (Fig.6A).

The same treatment with OD did not induce a significant variation in gene expression with respect to controls (data not shown). However, OD treatment induced an up-regulation of *RIPk2* gene (3.90-fold change) as also recorded for protein levels (Fig. 5B).

HD treatment induced a significantly up-regulation of the gene TNFRSF1A (8.10-fold change) after 2 h (Fig. 6B) but not TNFRSF1B. Interestingly HD induced an up-regulation of *Fas/FasL-Ligand System* with a 3.35 and 4,77 in fold change (Fig. 6B) which was not revealed with DD. HD also increased *FADD* gene expression (6.5-fold change, *caspase-3* (2.93-fold change) and *AIFM1* (6.19-fold change). (Fig. 6B).

### Cell cycle analysis

**Figure 7:**
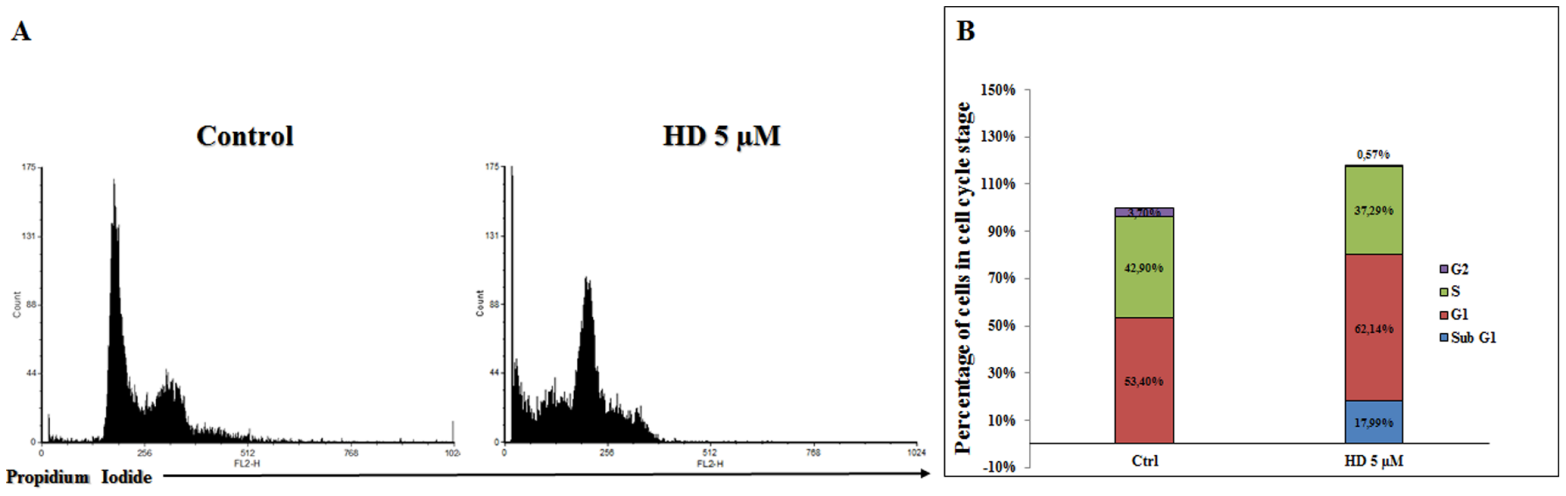
(A) Flow cytometric analysis of DNA content. Cells were exposed to 2-trans,4-trans-heptadienal (HD) 5 µM for 24 h. (B), The corresponding percentage of cells in each phase, obtained by ModFit LT Software.

We performed flow cytometry to analyze the effects of PUAs on the cell cycle of A549 cells. DD-exposed cells showed massive DNA fragmentation at 5 and 10 µM already after 24 h denoting rapid necroptosis which renders the identification of the stage at which cells were arrested difficult (Table 1). At lower concentrations (2 µM) only 15% of cells showed DNA fragmentation. At this concentration cells were arrested in G1/G2 after 48 h and 72 h treatment. In the case of OD, the number of cells in the S population were significantly reduced after 10 µM treatment (Table 1), suggesting a block in G1 and G2, with almost 50% cells showing DNA fragmentation. The same was true for 5 µM OD after 48 h and for 2 µM OD after 72 h (Table 1), suggesting a concentration-dependent effect. By contrast, HD-exposed cells showed an increase in the number of cells in either G1 or S at 5 µM after 24 h, suggesting that this is a threshold concentration probably involved in Fas death signaling (Fig. 7 and Table 1). These cell-cycle data support our findings regarding gene and protein expression that DD/OD and HD induce different death mechanisms in these cells.

**Table1:** Percetage of A549 cells treated with the PUAs 2-*trans*,4-*trans-*decadienal (DD), 2-*trans*,4-*trans*-octadienal (OD) and 2-*tran*s,4-*trans*-heptadienal (HD) in each cell cycle phase after times 0 and 24 h.

PUAs Concentration (µM)											
Time (h)	Cell Cycle Phase	Control	DD 10	DD 5	DD 2	OD 10	OD 5	OD 2	HD 10	HD 5	HD 2
**0**	**G1**	43.97	/	/	/	/	/	/	/	/	/
	**G2**	6.38	/	/	/	/	/	/	/	/	/
	**S**	49.65	/	/	/	/	/	/	/	/	/
	**Sub G1**	0.00	/	/	/	/	/	/	/	/	/
**24**	**G1**	53.40	0	0	51.65	65.5	52.18	51.8	59.77	62.14	47.55
	**G2**	3.7	0	0	0.95	27.42	8.19	8.83	9.77	0.57	2.51
	**S**	42.9	0	0	47.4	7.09	39.63	39.38	30.46	37.29	49.94
	**Sub G1**	0.01	0	0	15.42	49.89	26.59	1.82	0.32	17.99	0.4

## Discussion

Our results confirm that DD blocks the proliferation of A549 and COLO 205 cells at similar concentrations (2 and 5 µM) as previous studies using marine organisms [1], [3] and other cancer cell lines [15], [6]. We also confirm that this was the strongest of the three PUAs tested [25]–[27], at all time-intervals considered. However, after 48 h and 72 h HD becomes as strong as DD at 10 µM concentrations. OD was the least active of the three PUAs at all concentrations and time intervals tested. These results were confirmed by cell morphology analysis: nuclei stained with AO/EB showed occurrence of apoptosis after 48 h of treatment with DD and HD at higher concentration (10 µM). Both PUAs had stronger effects compared to the OD treatment. These findings have interesting implications from the ecological viewpoint considering that HD is the most common PUA produced by diatoms [24], and was considered until now as the most inactive of the three aldehydes having minor effects on grazer reproduction (e.g. [25]).

**Figure 8:**
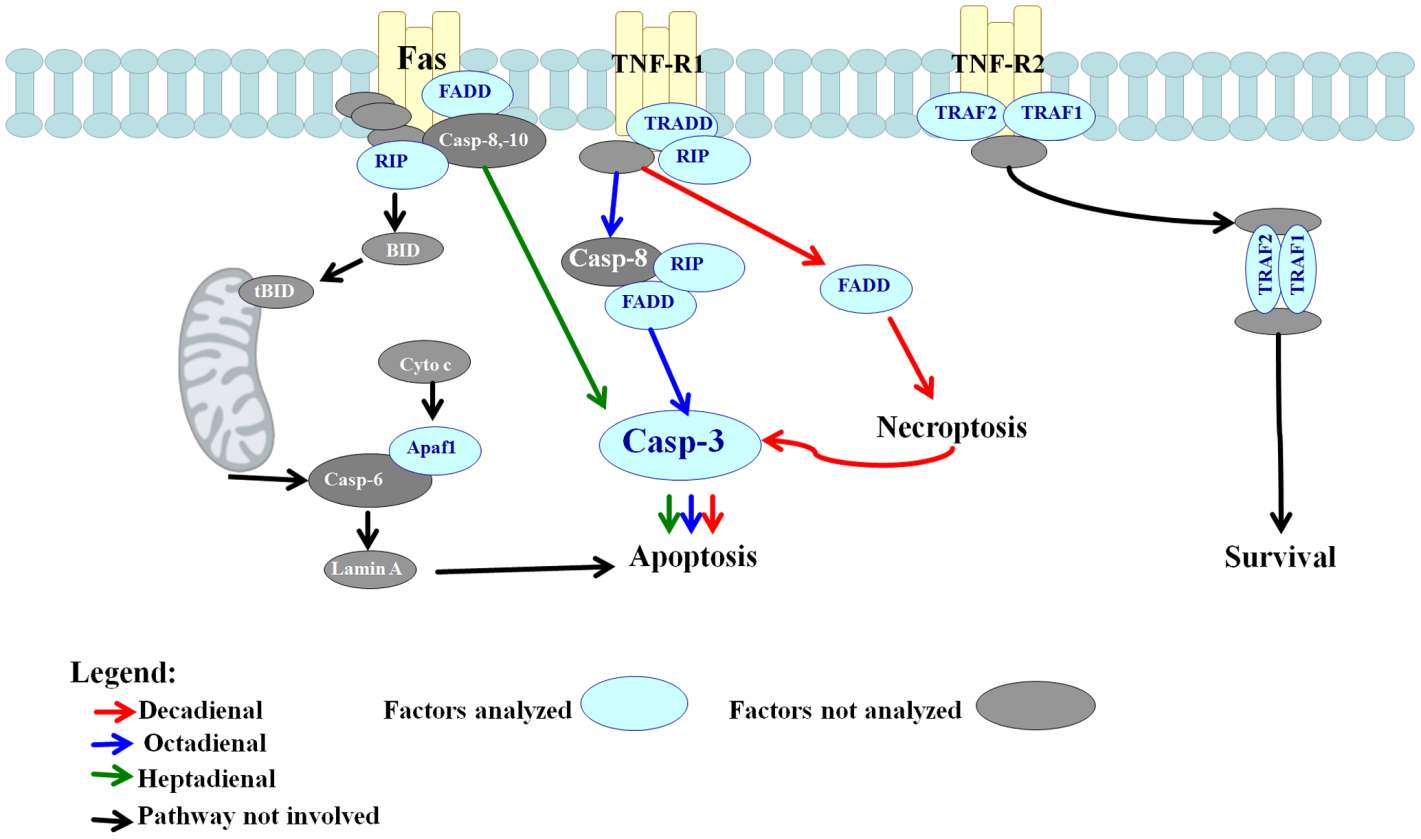
Schematic representation of pathways induced by 2-*trans*,4-*trans*-decadienal (DD), 2-*trans*,4-*trans*–octadienal (OD) and 2-*trans*,4-*trans*-heptadienal (HD) in A549 cell line. Black arrows indicate possible correlated pathways not involved in the response of cells to aldehyde treatment.

Another interesting finding in the present study regards the cell death (apoptosis) pathway and mechanism of signal transduction which differed among the three PUAs. A549 cells treated with all three PUAs showed a typical extrinsic apoptotic pathway [32]. As shown schematically in Fig. 8, cells treated with DD activated TNF-Receptor 1 (TNFR1) that in turn activated Fas Associated Death Domain (FADD) which was responsible for the caspase cascade after 24 h. This pathway did not involve expression of RIP indicating that the insult induced by DD was strong enough to directly activate necroptosis. The TNFR1/FADD/caspase pathway was also observed with OD, but only after 48 h. However, with OD there was also an increase in protein RIP expression levels that probably activated a survival pathway, consistent with the finding that OD causes less damage to the cell compared to DD (see Figs 1 and 2). Our results with OD are similar to those of Cheah and co-workers [33] who also found that another natural product, panduratin A, isolated from the Chinese medical herb *Boesenbergia rotunda,* induced apoptosis in the cancer cell line A549 along with the activation of a survival signaling pathway.

In contrast, cells treated with HD activated the receptor protein Fas that plays an important role in inducing early apoptosis mechanisms without activation of survival signals [34]. The absence of activation of TNFR1 indicates that HD causes a specific cell suicide compared to the other two PUAs. Fas in turn activated FADD which then triggers the same caspase signaling pathway as DD and OD without the involvement of RIP. In particular A549 cells treated with HD in the presence of caspase-3 inhibitor revealed a fast decrease in cell viability, probably due to the activation of AIFM1 as shown by gene expression analysis (Fig. 6B). When activated, this factor induces apoptosis in a caspase-independent manner. This is the first report of a marine natural product (HD) which induces an increase in Fas expression in A549 cells other than celastrol, extracted from the traditional Chinese medicinal herb, *Tripterygium wilfordii* Hook [35].

For all three aldehydes the increased expression of TNFR2 does not result in TRAF1 and TRAF2 activation, revealing the absence of these survival pathways. Thus, TRAF protein availability and function may regulate a cell survival checkpoint involving either a stress response on the one side or programmed cell death on the other [36]. The lack of activation or expression of Akt1, Akt2, PARP, and Apaf1 suggests that the mechanism of apoptosis induction by PUAs involves the extrinsic pathway in contrast to the other anticancer drugs that activate the intrinsic apoptotic pathway in this highly resistant cell line [37]–[39]. The particular effect of HD was also confirmed by cell-cycle data showing that whereas DD and OD arrested cells in the G1/G2 phase, HD blocked cells at the G2/S phase. On the other hand, BEAS-2B normal cells were not affected by any of the 3 PUAs tested indicating that that these aldehydes specifically target proliferating tumor cell lines and not somatic differentiated cells, supporting a previous study with DD on A1 mesenchymal neuronal cell line [6].

Our study provides a first insight into the strong effects of HD compared to DD which has been the model aldehyde used until now to understand the mechanism of PUA apoptosis induction in invertebrates (e.g. [27]). By activating Fas and therefore a direct suicide program without any survival mechanisms implies that the damaging effects of HD on grazers are irreversible. Furthermore, the strong effect observed in highly proliferating cells such as A549 may explain why damaging effects seem to mainly target invertebrate female gonads and early embryos [29], [40]. Future efforts in toxicological studies should therefore be directed to better clarify the negative effects of this molecule on grazers since this is the PUA which will cause the most damaging effects during diatom blooms at sea [41]. Given the importance of diatom blooms in nutrient-rich aquatic environments, our results have important implications for understanding the cellular mechanisms underlying the responses of marine planktonic and benthic organisms to toxic PUA exposure. Our study also proposes a protocol using a resistant cancer cell line for testing bioactive antiproliferative compounds from marine microalgae for biotechnological applications.
